# Supplementary material for: Participation of HHIP Gene Variants in COPD Susceptibility, Lung Function, and Serum and Sputum Protein Levels in Women Exposed to Biomass-Burning Smoke
Source: Diagnostics (Basel). 2020 Sep 23;10(10):734. doi: 10.3390/diagnostics10100734 (PMC7598157; doi:10.3390/diagnostics10100734)
Supplement: Supplementary file 1 [file diagnostics-10-00734-s001.pdf]

Supplementary materials

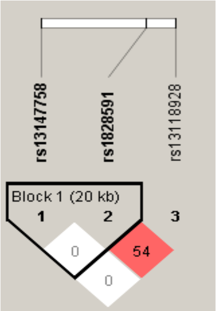

Haplotypes	COPD-BS	BBES	p	OR	CI 95%
AA	243	669	0.07	1.25	0.98 – 1.60
GG	99	305	0.78	0.96	0.73 – 1.25
GA	19	80	0.18	0.69	0.41 – 1.16
AG	11	60	0.06	0.53	0.27 – 1.02

Figure S1. Haplotypes of the rs13147758 and rs18285918 in the *HHIP* gene. COPD-BS: COPD related to biomass-burning exposure; BBES: Biomass-burning smoke-exposed subjects: $p<0.05$ statistical significance; OR: Odds ratio; CI 95%: 95% confidence interval; showing r^2 values among SNPs.

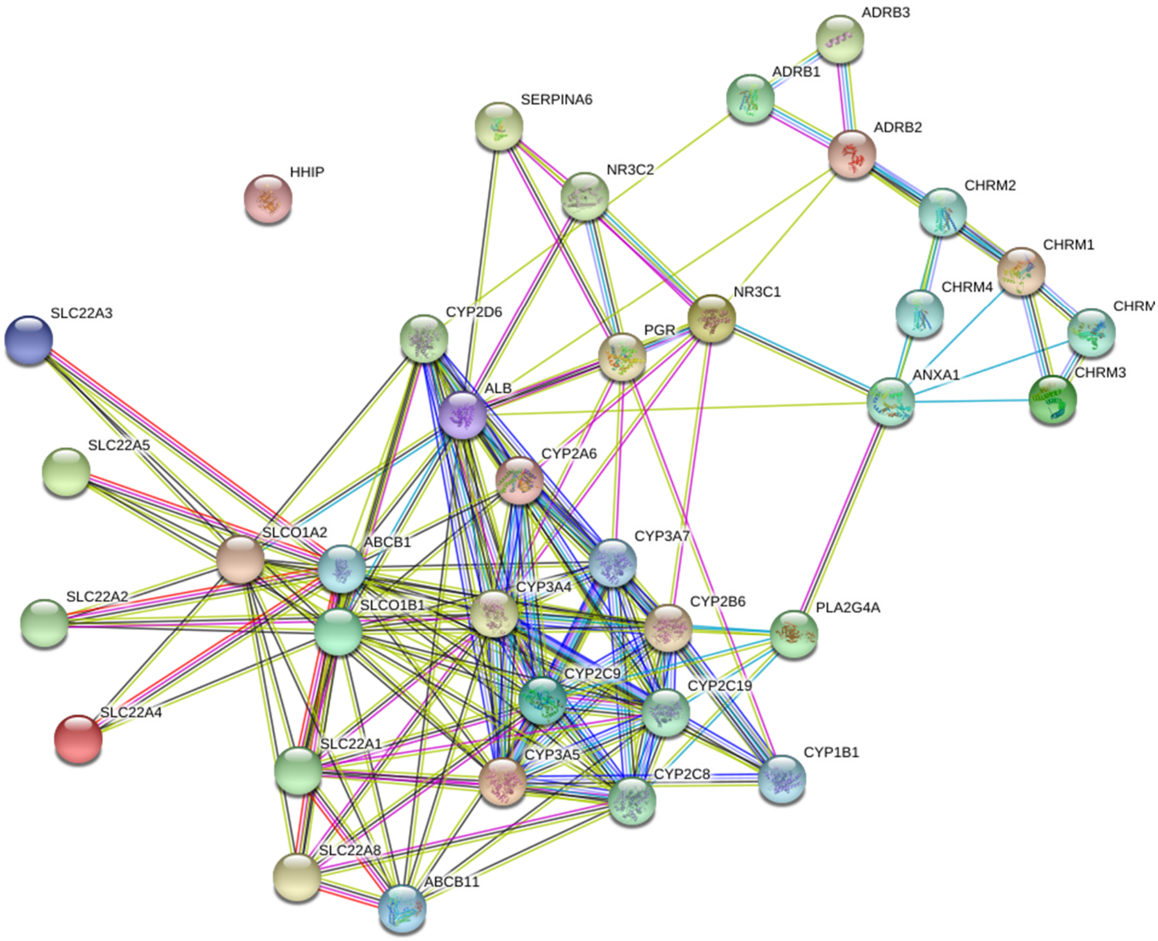

Figure S2. In silico analysis from the metabolism of drugs in the treatment of COPD, and the interaction of HHIP with pharmacological treatment.

Table S1. Genotype frequencies comparison among COPD patients, according to G2 vs. G1 groups.

SNP Genotype	G2 n= 37	G2 %	G1 n= 149	G1 %	p	OR	CI 95%
**rs13147758**							

**AA**	17	45.95	70	46.98	1	0.95	0.46 – 1.97-
**AG**	16	43.24	64	42.95	1	1.01	0.48 – 2.09
**GG**	4	10.81	15	10.07	1	1.08	0.33 – 3.47
**rs13118928**							
**AA**	17	45.95	71	47.65	1	0.93	0.45 – 1.92
**AG**	18	48.64	68	45.64	0.85	1.12	0.54 – 2.32
**GG**	2	5.41	10	6.71	1	0.79	0.16 – 3.79
**rs1828591**							
**AA**	21	56.76	69	46.31	0.27	1.52	0.73 – 3.14
**AG**	15	40.54	62	41.61	1	0.95	0.45 – 1.99
**GG**	1	2.70	18	12.08	0.12	0.20	0.02 – 1.56

SNP: Single Nucleotide Polymorphism; G1 (GOLD I + II); G2 (GOLD III + IV); p statistically significant (<0.05); OR odds ratio; 95% CI confidence interval of 95%.

Table S2. Characteristics of selected women for serum protein levels analysis.

	**COPD-BS (n= 40)**	**BBES (n= 40)**	**p**
Age (Years)	71.50 (56 - 86)	64 (51 - 87)	0.003
BMI	25.43 (19.3 - 39.4)	27.30 (20.7 - 37.8)	0.043
BBEI	312 (140 - 828)	290 (108 - 1050)	0.17
FEV_1 (%)	46.50 (18 - 79)	104 (69 - 135)	<0.001
FVC (%)	69.50 (43 - 146)	95.50 (65 - 134)	<0.001
FEV_1/FVC (%)	50 (24 - 69)	82.80 (72 - 138)	<0.001

COPD-BS: exposed biomass with COPD; BBES: exposed to biomass-burning smoke: p<0.05 statistical significance; BMI: body mass index; BBEI: Biomass-burning smoke exposure index, FEV_1: forced expiratory volume in the first second; FVC: forced vital capacity. The median and min. - max values are shown.

Table S3. Demographical, clinical, exposition and lung function of exposed to biomass-burning smoke of the subgroup for protein levels in supernatant sputum.

	**COPD-BS (n= 20)**	**BBES (n= 20)**	**p**
Age (years)	72 (55 - 88)	61.50 (50 - 82)	0.003
BMI	25.7 (17.7 - 41.4)	28.7 (19.8 - 39.2)	0.041
BBEI	470 (270 - 1050)	360 (120 - 720)	0.076
FEV_1 (%)	68.50 (16 - 96)	104 (74 - 159)	<0.001
FVC (%)	81 (38 - 122)	110 (81 - 168)	<0.001
FEV_1/FVC (%)	60 (32 - 75.20)	86 (74.90 - 121)	<0.001

COPD-BS: exposed biomass with COPD; BBES: exposed to biomass-burning smoke: p<0.05 statistical significance; BMI: body mass index; BBEI: Biomass-burning smoke exposure index, FEV_1: forced expiratory volume in the first second; FVC: forced vital capacity. The median, minimum, and maximum values are shown.

Table S4. General characteristics of SNPs included in the analysis.

**SNP**	**Position/ (Strand)**	**Ancestral allele**	**Change**	**MAF**	**MXL**	**Gene location**
rs13118928	144565237 (FWD)	A	A/G	G=0.30	G=0.28	Intron
rs1828591	144559628 (FWD)	A	A/G	G=0.41	G=0.30	Intron
rs13147758	144539078 (FWD)	A	A/G	G=0.29	G=0.32	Intron

SNP: single nucleotide polymorphism; FWD: forward chain; MAF: minor frequency allele; MXL: Mexican Ancestry in Los Angeles, California; HWE: Hardy-Weinberg Equilibrium.
